# Health Professionals in a COVID-19 Reference Hospital: Post-traumatic Stress Disorder (PTSD) Levels and Their Associations With Psychological Resilience and Quality of Life

**DOI:** 10.7759/cureus.22473

**Published:** 2022-02-21

**Authors:** Dimitra Lekka, Konstantina Orlandou, Christos Pezirkianidis, Aikaterini Roubi, Athanasios Tsaraklis, Constantinos Togas, Sofia Mpoulougari, Frosyna Anagnosti, Dimitra Darahani, Anastasios Stalikas

**Affiliations:** 1 Department of Psychiatry, Sotiria Thoracic Diseases Hospital of Athens, Athens, GRC; 2 Department of Psychology, Panteion University of Social and Political Sciences, Athens, GRC; 3 Department of Psychology, Panteion University, Athens, GRC; 4 Departement of Dermatology, Sotiria Hospital, Athens, GRC; 5 Department of Administration, Sotiria Thoracic Diseases Hospital of Athens, Athens, GRC; 6 Emergency Department, Sotiria Thoracic Diseases Hospital of Athens, Athens, GRC; 7 Department of Cardiology, Sotiria Thoracic Diseases Hospital of Athens, Athens, GRC

**Keywords:** healthcare professionals, greece, quality of life, ptsd, covid-19

## Abstract

Introduction

The coronavirus disease 2019 (COVID-19) pandemic has affected the quality of life of both the general population and health professionals and has increased the levels of psychopathology among them. The present study aims to map the levels of post-traumatic stress disorder (PTSD), psychological resilience, and quality of life of healthcare professionals who work in a COVID-19 reference hospital in Athens, Greece, one year after the onset of the pandemic. Also, this study focuses on investigating the relationships among the study variables and demographics and examining possible mediating effects.

Methods

The sample consisted of 400 health professionals from Sotiria Hospital, of whom 102 were men. Participants were asked to complete the Post-Traumatic Stress Scale, Connor-Davidson Resilience Scale, and the WHO Quality of Life Questionnaire. The survey was conducted from May to July 2021.

Results

The findings show statistical differences in resilience levels regarding marital status and employee education. Also, 13.5% of the staff reported significant PTSD levels, which relate to low levels of psychological resilience and every pillar of quality of life.

Conclusions

Thus, research findings indicate that resilience levels could have a protective effect on the development of PTSD symptoms. Therefore, the design of group interventions that aim at building health workers’ resilience will be discussed.

## Introduction

In December 2019, an outbreak of unprecedented pneumonia caused by the virus severe acute respiratory syndrome coronavirus 2 (SARS-CoV-2) was reported in Wuhan and has led to a global pandemic [1]. The worldwide transmission of the pandemic has been found to cause significant effects on the mental health of individuals, such as the onset of psychiatric syndromes in healthy individuals but also the worsening or recurrence of symptoms in individuals already experiencing a psychiatric disorder [2], including depression, anxiety disorders, panic attacks, somatic disorders, post-traumatic stress disorder (PTSD), psychosis, and suicide [3].

Research findings indicate the impact of the coronavirus disease 2019 (COVID-19) pandemic on the physical and mental health of the general population [4]. For instance, there is a 25% increase in depression levels in the general population worldwide, which is almost seven times higher than in the pre-COVID-19 era [5]. Many researchers emphasize that the most important factor related to the general population's mental health deterioration is social distancing and isolation [6]. Those factors also impact the psychological health of high-risk groups, like pregnant women, health professionals, and children [7]. A study in Greece showed a 37.6% increase in sleep disorders, especially in women, city residents, and the elderly. In addition, the same study showed that people who were strongly concerned about a possible COVID-19 infection experienced increased levels of loneliness [8].

Several factors play a protective role against psychopathology during the COVID-19 pandemic, e.g., the social capital, total personal resources, sufficient coping abilities, and the supportive network of friends, parents, and acquaintances [9]. Furthermore, the psychological resilience of individuals seems to mediate the relationship between the impact of stressful events related to the virus and the levels of psychopathology [10]. Thus, researchers suggest focusing on programs to enhance psychological resilience or social relationships as antidotes to the negative effects of COVID-19-related loneliness [11].

It is well known that trauma, chronic adversity, and stressful life events impact the function and structure of the brain and are likely to lead to the development of PTSD [12]. Individuals that face PTSD symptoms report lower levels of quality of life [13] and psychological resilience [14]. Healthcare workers involved in the COVID-19 pandemic have been identified as a high-risk group that has already reported symptoms of PTSD, depression, insomnia, grief, and distress [15]. Furthermore, in a prior study that was conducted among the healthcare workers in Sotiria Hospital, Athens, it was found that healthcare workers dehumanize the patients mechanistically [16]. A literature review carried out by Danet [17] underlined that the levels of depression, stress, sleep disturbance, and burnout among healthcare workers in the USA and Europe were higher in frontline workers, nurses, and females.

The majority of individuals who are exposed to difficult situations do not develop PTSD as a result of high psychological resilience levels [12]. Moreover, research findings indicate that, when facing difficulty, PTSD levels, directly and indirectly, affect the quality of an individual’s life. However, resilience mediates this relationship and acts protectively on quality of life levels [12]. Furthermore, a study that took place in Sotiria Hospital in 2021 revealed that the quality of life of healthcare professionals has been affected by COVID-19 [18]. Substantial evidence supports the effectiveness of resilience to preserve psychological and mental health among hospital workers during the current pandemic [19].

According to a survey conducted in Greece, criteria for a PTSD diagnosis were met in 15% of health workers [20]. Moreover, a comparison between hospital staff working in COVID-19 clinics and other units revealed that the former reported higher levels of PTSD, and the variables of gender and marital status significantly predicted the PTSD symptoms [15].

Therefore, this research aimed to study PTSD, quality of life, and resilience on healthcare professionals in a tertiary referral hospital of COVID-19 in Greece, to design interventions that will contribute to relief the staff and increase their well-being levels. The research questions of the present study are as follows: (1) What are the levels of PTSD, resilience, and quality of life of health professionals in Greece? (2) What are the differences between men and women in PTSD, resilience, and quality of life levels of health professionals in Greece? (3) Are there significant differences in the levels of resilience and quality of life among health professionals that face high and low PTSD levels? (4) What is the correlation between PTSD, resilience, and quality of life levels of health professionals? (5) Do PTSD levels predict health professionals' resilience and quality of life?

We assume that healthcare professionals with high levels of psychological resilience report lower levels of PTSD and higher levels of quality of life.

## Materials and methods

Participants

The sample consisted of 400 healthcare workers working in the Thoracic Diseases General Hospital "Sotiria" in Athens, Greece. Regarding the specialty of the study participants, 69 participants were doctors (17.3%), 211 were nurses (52.8%), 57 were administrative staff (14.2%), 16 were scientific staff (4%), and 47 were from other specialties (11.8%). Also, of the participants, 102 were men (25.5%) and 298 were women (74.5%). With regard to age, 76 (19%) participants were aged between 20 and 30 years, 122 were between 31 and 40 (30.5%) years, 119 were between 41 and 50 (29.8%) years, and 83 were between 51 and 67 (20.8%). Concerning the level of education, 117 participants were graduates of secondary school/lyceum (29.3%), 175 were graduates of university (43.8%), and 108 had a master's degree or a PhD (27%). Regarding their marital status, 147 participants were single (36.8%), 218 were married/partnered (54.5%), 34 were divorced (8.5%), and one participant was a widow/widower (0.3%).

Measures

The World Health Organization Quality of Life, Brief Version (WHOQOL-BREF) is a quality of life assessment created by the WHO [21]. It measures four aspects of an individual’s quality of life: physical health, psychological health, social relationships, and environment. Thus, it depicts the multidimensional nature of the quality-of-life structure [21]. The WHOQOL-BREF contains 26 items. The scores of the four domains (physical health, psychological health, social relationships, and environment) are being converted according to the WHOQOL-BREF manual, to a 0-100 scale [22], with higher scores denoting higher levels of quality of life. The WHOQOL-BREF has been validated to the Greek population and showed satisfactory reliability and validity. Concerning its reliability levels, the Cronbach’s alpha coefficients in our study are adequate, i.e., α = 0.90 for the total score, α = 0.81 for the physical health subscale, α = 0.69 for the psychological health subscale, α = 0.78 for the social relationships subscale, and α = 0.80 for the environment subscale.

The Connor-Davidson Resilience Scale (CD-RISC) is a short self-report questionnaire for measuring mental durability and consists of 25 items, which are scored in a five-point Likert rating scale (0-4), with the highest scores reflecting greater resilience. The scale according to Connor and Davidson [14] measures five factors, which are: (a) personal competence, high criteria, and perseverance (eight items); (b) confidence in personal instinct, long-suffering in negative mood, as well as strengthening effect of stress (seven items); (c) positive acceptance of change and secure relationships (five items); (d) control (three items); and (e) intellectual influences (two items). The scale shows a satisfactory convergent and divergent validity and good internal consistency levels (α = 0.89) and also good test-retest reliability (r = 0.87) [23]. The CD-RISC includes inter alia questions that reflect the dimensions of the concept of "hardiness," such as control, commitment, and challenge [14]. The Cronbach’s alpha coefficient in our study is α = 0.94 for the total score.

The Post-Traumatic Stress Disorder Checklist-Civilian version (PCL-C) was developed by Weathers et al. [24] and it includes 17 items that correspond to the main symptoms of PTSD based on the Diagnostic and Statistical Manual of Mental Disorders, fourth edition (DSM-IV). Also, the checklist includes items measuring the manifestation of indicative symptoms of PTSD in the past as well as the existence of psychotic symptomatology. The total score of PCL-C (i.e., the sum of scores for the 17 items) ranges from 17 to 85. With this in mind, previous studies have suggested that scores of 45-50 provide the best discrimination between cases and non-cases of PTSD. In previous studies, the scale demonstrated high levels of internal consistency ranging from α = 0.94 [25] to α = 0.97 [24]. Concerning its reliability level, Cronbach’s alpha coefficient in our study was adequate, α = 0.95 for the total score.

Procedure

The study was performed at a tertiary referral hospital of COVID-19 in Greece after approval from the Clinical Research Committee (approval number: 9587/5-4-21). Thoracic Diseases General Hospital "Sotiria" is well-known as the largest pulmonary center in Greece. In the last years, it has been transformed into a general hospital after the integration of pathology and surgery clinics. The hospital received the first case of SARS-CoV-2 in February 2020, and until October 2021, it remained a hospital specializing in COVID-19 cases.

For the selection of the sample, the technique of convenience sampling has been chosen. The sample was drafted from May 2021 to July 2021. The questionnaire was administered in person to the health professionals, who volunteered to take part in the study. The questionnaire explains both the anonymity and the right to voluntarily draw out from the study, as well as the objectives of the study and the procedures to be followed during the questionnaire.

Data analysis

Data analysis was performed using SPSS version 26 (IBM Corp., Armonk, NY) and statistical significance was set to 5%. The assumption of normality and homoscedasticity of variances were examined to determine whether to use parametric or non-parametric tests. Results were acquired by means of descriptive statistics, t-test, Pearson’s correlation, and regression. The critical level of p < 0.05 of Kolmogorov-Smirnov statistics was analyzed, as well as the levels of asymmetry and kurtosis. From these analyses, it was deduced that the data did not follow a normal distribution; however, it was decided to use parametric tests because the distribution of the data was close to normal distribution.

## Results

An independent sample t-test was utilized to examine possible differences among participants with and without PTSD symptoms regarding the quality of life aspects. The results showed statistically significant differences in every aspect of quality of life. More specifically, differences found on the levels of physical health (t(398) = 6.50, p < 0.001, d = 0.99) were with a big effect size, psychological health (t(398) = 6.56, p < 0.001, d = 0.97) were with a big effect size, social relationships (t(398) = 6.89, p < 0.001, d = 0.96) were with a big effect size, and environment (t(398) = 3.79, p < 0.001, d = 0.52) were with a medium effect size. All differences indicate that individuals with no PTSD symptoms report better quality of life levels in comparison with those with PTSD symptoms (Table 1).

**Table 1:** Mean differences on quality of life variables among participants with (n = 52) and without (n = 348) PTSD symptoms. PTSD, post-traumatic stress disorder.

Variables	Groups	M (SD)	t	df	p	d
Physical health	No PTSD	3.76 (0.50)	6.50	398	0.000	0.99
	PTSD	3.29 (0.47)				
Psychological health	No PTSD	3.71 (0.50)	6.56	398	0.000	0.97
	PTSD	3.22 (0.51)				
Social relationships	No PTSD	3.80 (0.59)	6.89	398	0.000	0.96
	PTSD	3.19 (0.68)				
Environment	No PTSD	3.27 (0.57)	3.79	398	0.000	0.52
	PTSD	2.93 (0.70)				

It is important to mention that analysis of variance (ANOVA) was used to examine the possible resilience levels with the demographic characteristics and then to focus on those who were statistically significant. The results concerning the resilience levels of marital status groups indicate the existence of statistically significant differences (F(3,396) = 3.23, p = 0.02, η2 = 0.20) with a big effect size. The participants in a relationship reported the highest resilience levels (M = 3.60, SD = 0.62). Moreover, regarding the educational level of the participants, statistically significant differences in resilience were found as well (F(2, 397) = 4.90, p = 0.01, η2 = 0.24) with a big effect size. The participants with master's degree/PhD reported the highest resilience levels (M = 3.68, SD = 0.53) (Table 2). The rest of the demographic characteristics did not display a significant effect on resilience and PTSD.

**Table 2:** Mean differences in resilience levels among participants with different marital statuses and educational levels.

Variables	n	M	SD	F	p	η^2^
Marital status						
Single	147	3.42	0.70	3.23	0.02	0.20
Married/partnered	218	3.60	0.62			
Divorced	34	3.27	1.05			
Widow/widower	1	3.84	-			
Level of education						
Secondary school/lyceum	117	3.44	0.77	4.90	0.01	0.24
University graduates	175	3.43	0.72			
Master/PhD	108	3.68	0.53			

Resilience correlates significantly positively with all dimensions of quality of life and is significantly negatively correlated with PTSD (Table 3).

**Table 3:** Relationship (Pearson's r) between resilience, PTSD, and quality of life. ** Correlation is significant at 0.01 level (two-tailed). PTSD, post-traumatic stress disorder.

	Resilience	PTSD	Physical health	Psychological health	Social relationships	Environment
Resilience		−0.25**	0.26**	0.29**	0.32**	0.23**
PTSD	−0.25**		−0.55**	−0.50**	−0.46**	−0.32**
Physical health	0.26**	−0.55**		0.68**	0.53**	0.49**
Psychological health	0.29**	−0.50**	0.68**		0.59**	0.34**
Social relationships	0.32**	−0.46**	0.53**	0.59**		0.43**
Environment	0.23**	−0.32**	0.49**	0.34**	0.43**	

From the regression analysis, it seems that resilience contributes significantly positively to the prediction of social relationships (p = 0.006), while in the rest (physical health, psychological health, environment, and PTSD), it does not contribute (p > 0.05).

## Discussion

After two years of the pandemic, it has become apparent worldwide that the healthcare systems have largely failed to successfully manage the SARS-CoV-2. It is observed that despite the advent of vaccines, the pressure on healthcare systems has not been greatly reduced, mainly due to the lack of funding from governments in the field of health, with the result that healthcare workers choose to leave their jobs due to the exhaustion they feel in a physical and emotional level.

A study conducted on the healthcare workers of a tertiary hospital in Wuhan confirms the findings of or study that marital status and gender do not affect PTSD levels. Also, these two surveys agree that the specialty of each employee did not seem to predict the occurrence of PTSD [26].

In addition, a survey in a hospital in Norway showed that 28.9% of healthcare workers presented with clinical and subclinical symptoms of PTSD at a time when in the “Sotiria” hospital, this percentage increased to 13.5% [27]. From this, we can assume that the fact that the staff recruited during the pandemic had previously worked in intensive care units at private hospitals and had relevant experience was one of the reasons for the non-occurrence of a very high percentage of PTSD in this population. This is confirmed by a research finding that showed that nurses who had increased levels of resilience were less likely to experience PTSD [28].

More specifically, in terms of resilience, our research showed that marital status and educational level significantly affect resilience levels. In fact, a corresponding survey conducted on the general population in Greece showed that people who had a relationship score higher on its scale [29]. However, in our research, it was illustrated that gender and age do not have a statistically significant effect on resilience, a finding that is consistent with some research findings, while with some others, it contradicts [29,30].

In addition, a survey conducted among Sotiria Hospital staff found that the young ones and the males exhibited a better quality of life and suggested interventions such as mindfulness and setting up teams to share experiences in supporting staff during the pandemic [18]. Also, another research at the same hospital found that health professionals mechanistically dehumanize the patient, while secure attachment acts protectively against self-dehumanization [16]. This is an important finding especially for the period we are going through, as the provision of human care to the hospitalized patient and the non-self-dehumanization of the staff is more imperative than ever for the effective management of the pandemic.

Our research naturally faced some limitations worth mentioning. Firstly, in the category of marital status, there was only one widow, so we cannot draw safe conclusions, and for this reason, a larger sample is needed. Secondly, women in the sample (N = 298) are significantly more than men (N = 102) and in the category of specialty, the scientific staff was very few (N = 16). Furthermore, the history of psychiatric disorders was not examined.

## Conclusions

It appears from the findings that healthcare workers need interventions to enhance resilience, focused on the here and now (mindfulness). Interventions are still needed in working groups, especially for those who are single or divorced and generally for people who do not have a supportive framework, as 13.5% of the hospital staff have PTSD. Finally, it would be fruitful to organize central planning so that those who work in hospitals with COVID-19 cases can rest.
